# P-237. Impact of Acute Malaria on Viral Load Among People with HIV: A Multi-Site Prospective Cohort Study in Sub-Saharan Africa

**DOI:** 10.1093/ofid/ofaf695.459

**Published:** 2026-01-11

**Authors:** Lauren Sweet, Seth Frndak, Nicole Dear, Kartavya J Vyas, Hannah Kibuuka, John Owuoth, Valentine Sing’oei, Jonah K Maswai, Reginald R Gervas, Abdulwasiu Tiamiyu, Zahra Parker, Trevor A Crowell, Julie A Ake, Neha Shah, Joseph Yabes

**Affiliations:** Brooke Army Medical Center, San Antonio, TX; Henry M Jackson Foundation, Bethesda, Maryland; Michigan State University, Grand Rapids, Michigan; U.S. Military HIV Research Program, Walter Reed Army Institute of Research, Bethesda, Maryland; Makerere University Walter Reed Project, Kampala, Kampala, Uganda; Henry M. Jackson Medical Research International, Kisumu, Western, Kenya; HJF Medical Research International, Kisumu, Western, Kenya; Walter Reed Army Institute of Research - Africa, Kericho, Rift Valley, Kenya; HJF Medical Research International, Tanzania, Mbeya, Mbeya, Tanzania; HJF Medical Research International, Kisumu, Western, Kenya; U.S. Military HIV Research Program, Walter Reed Army Institute of Research AfricaHenry M. Jackson Foundation for the Advancement of Military Medicine, Lagos, Lagos, Nigeria; Henry M. Jackson Foundation for the Advancement of Military Medicine, Bethesda, Maryland; Walter Reed Army Institute of Research, Silver Spring, Maryland; U.S. Military HIV Research Program, CIDR, Walter Reed Army Institute of Research, Bethesda, Maryland; Brooke Army Medical Center, San Antonio, TX

## Abstract

**Background:**

Characterization of malaria episodes among people with HIV (PWH) is lacking since shifts to HIV test-and-treat and adoption of integrase-strand inhibitors. Improvements in HIV care may have minimized previously seen impacts of malaria on HIV outcomes.

**Table 1.**
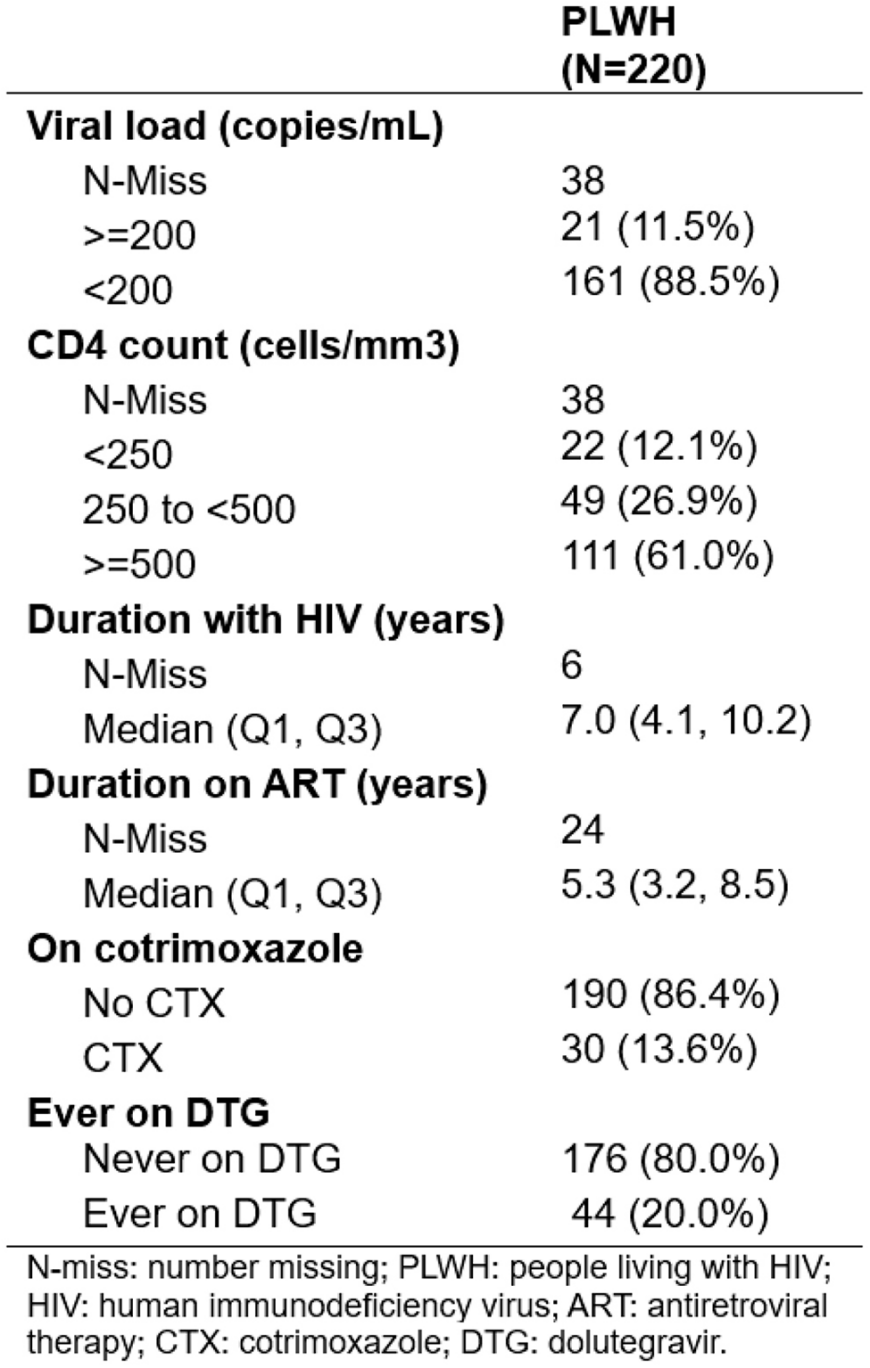
Clinical characteristics of PLWH participants at closest visit to first positive malaria test

**Figure 1.**
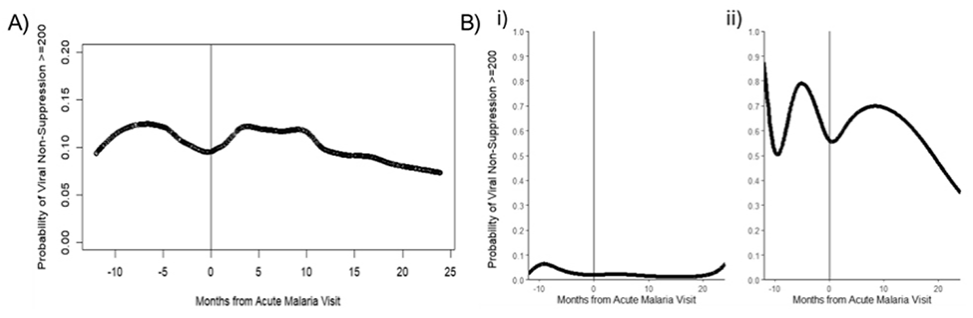
A) Predicted probability of viral non-suppression (>=200 copies/mL) across months from acute malaria visit. B) Growth Mixture modeling of the 2 identified clusters. i) consistent suppressors, and ii) non-suppressors and the probability of viral non-suppression prior-to and after the malaria event. Malaria event is noted at time “0”.

**Methods:**

PWH and people without HIV (PWoH) from Uganda, Kenya, Tanzania and Nigeria who enrolled in the African Cohort Study (AFRICOS) in 2013-2023 were considered (n=4,262). Alternate infectious diagnoses were excluded and participants with a febrile visit and a positive malaria smear or PCR were included (n=584). Demographic and clinical characteristics at prior visit closest to positive malaria smear were compared. Complete case analyses were utilized for missing data. Growth mixture modeling (GMM) of PWH by viral load (VL) was performed before and after an acute malarial episode (AME) to elucidate trajectories of VL around the episode. Clusters were further compared by clinical parameters including ART status, and CD4 count.

**Table 2.**
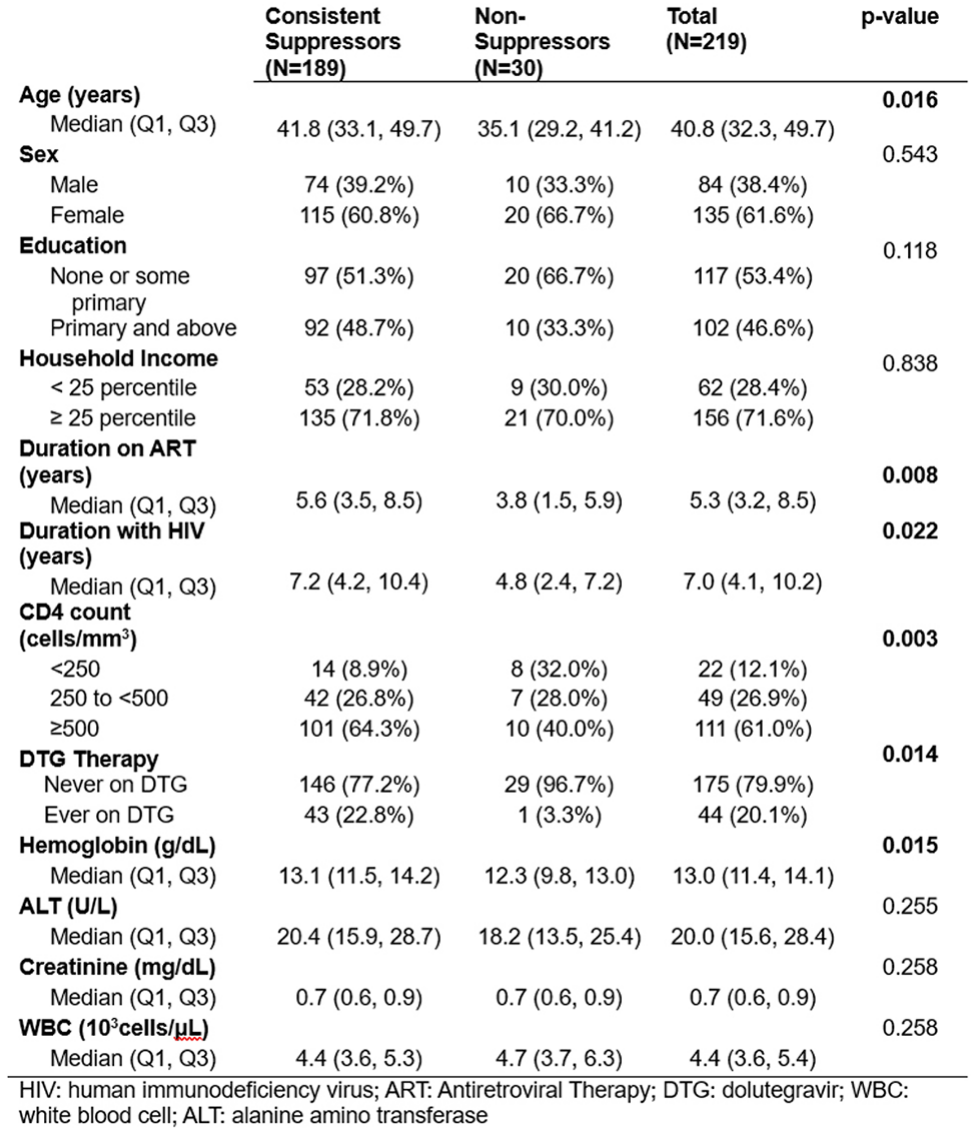
Clinical characteristics of participants at visit closest to acute malaria visit, by cluster

**Results:**

A total of 220/468 (47.0%) PWH and 74/116 (63.8%) PWoH tested positive during the study period. At first positive test, PWH had significantly higher ALT (median [M]=20.1U/L, interquartile range [IQR]=15.7-28.7 vs. M=16.0, IQR=12.2-22.9) and lower white blood cell count (WBC; M=4.4 x 103 cells/μL, IQR=3.6-5.4 vs. M=4.9, IQR=4.5-6.3) than PWoH. Most PWH positive for malaria were virally suppressed < 200 copies/mL (88.5%) and on ART for 5 years (M=5.3; Table 1). The GMM of PWH (n=219 with 2 timepoints) revealed 2 clusters: consistent VL suppressors (VS; n=189, 86.3%) and VL non-suppressors (VNS; n=30, 13.6%). Probability of VL non-suppression increased 6 months before and 3 months after AME (Figure 1). VNS were younger (M=35.1 years), had less time since HIV onset and on ART (M=3.8 years), less likely to be ever on dolutegravir (DTG), and had lower CD4 counts compared to VS (60% vs 35.7% with < 500 cells/mm3)(Table 2).

**Conclusion:**

PWH had generally comparable baseline clinical parameters to PWoH at acute malaria visit. Among PWH, a longitudinal cluster of VNS emerged among younger and newly HIV-diagnosed individuals not on DTG. VNS remains low overall, showing the benefits of continued HIV care engagement. Future studies are needed to elucidate facets of care surrounding HIV outcomes and malaria.

**Disclosures:**

All Authors: No reported disclosures

